# Association of Aortic Compliance and Brachial Endothelial Function with Cerebral Small Vessel Disease in Type 2 Diabetes Mellitus Patients: Assessment with High-Resolution MRI

**DOI:** 10.1155/2016/1609317

**Published:** 2016-07-20

**Authors:** Yan Shan, Jiang Lin, Pengju Xu, Mengsu Zeng, Huandong Lin, Hongmei Yan

**Affiliations:** ^1^Department of Radiology, Zhongshan Hospital, Fudan University and Shanghai Institute of Medical Imaging, Shanghai 200032, China; ^2^Department of Endocrinology, Zhongshan Hospital, Fudan University, Shanghai 200032, China

## Abstract

*Objective*. To assess the possible association of aortic compliance and brachial endothelial function with cerebral small vessel disease in type 2 diabetes mellitus (DM2) patients by using 3.0 T high-resolution magnetic resonance imaging.* Methods*. Sixty-two clinically confirmed DM2 patients (25 women and 37 men; mean age: 56.8 ± 7.5 years) were prospectively enrolled for noninvasive MR examinations of the aorta, brachial artery, and brain. Aortic arch pulse wave velocity (PWV), flow-mediated dilation (FMD) of brachial artery, lacunar brain infarcts, and periventricular and deep white matter hyperintensities (WMHs) were assessed. Pearson and Spearman correlation analysis were performed to analyze the association between PWV and FMD with clinical data and biochemical test results. Univariable logistic regression analyses were used to analyze the association between PWV and FMD with cerebral small vessel disease. Multiple logistic regression analyses were used to find out the independent predictive factors of cerebral small vessel disease.* Results*. Mean PWV was 6.73 ± 2.00 m/s and FMD was 16.67 ± 9.11%. After adjustment for compounding factors, PWV was found significantly associated with lacunar brain infarcts (OR = 2.00; 95% CI: 1.14–3.2; *P* < 0.05) and FMD was significantly associated with periventricular WMHs (OR = 0.82; 95% CI: 0.71–0.95; *P* < 0.05).* Conclusions*. Quantitative evaluation of aortic compliance and endothelial function by using high-resolution MRI may be potentially useful to stratify DM2 patients with risk of cerebral small vessel disease.

## 1. Introduction

Type 2 diabetes mellitus (DM2) can cause many cardiovascular complications including ischemic stroke which is a leading cause of death [1, 2]. Arterial endothelial dysfunction and arterial compliance abnormalities are early arterial changes in DM2 patients which occur earlier than structural abnormalities of vessel walls and clinical onset of cardiovascular complications [3–5]. Studies have shown that arterial stiffening is a strong predictor of future cardiovascular events and all-cause mortality and an independent predictor of fatal stroke [6, 7].

Cerebral small vessel disease is one of the common cardiovascular complications in DM2 patients [8]. There is report that the stroke risk in patients with DM2 is two to five times higher than that in patients with normal glucose [9]. If an association of arterial endothelial dysfunction and arterial compliance abnormalities with cerebral small vessel disease could be established, this would support the significance of measuring arterial endothelial dysfunction and arterial compliance abnormalities for early prediction, stratification, and prevention of this cardiovascular complication in DM2 patients. It has been reported that PWV is independently associated with cerebral small vessel disease in patients with type 1 DM [10]. Another study has shown that FMD is associated with poor prognosis in patients with ischemic stroke [11]. Compared with DM1, DM2 has higher incidence; furthermore, its prevalence is increasing rapidly not only in developed countries but also in developing countries [12]. To our knowledge, there has been no report so far about association between aortic compliance and brachial endothelial function with cerebral small vessel disease in DM2 patients by using magnetic resonance imaging (MRI).

The advantages of MRI include noninvasiveness, being without radiation, high soft tissue resolution, a large field of view, and being less operator-dependent. The previous study has demonstrated that high-resolution MRI can provide superior image quality and reproducibility for the assessment of arterial compliance and endothelial function of the central and peripheral arteries during a single examination [13]. In addition, MRI is superior in the evaluation of lacunar brain infarcts and white matter injuries resulted from cerebral small vessel disease [14].

Therefore, we attempt to assess the possible association between aortic compliance and endothelial function with cerebral small vessel disease in DM2 patients by using 3.0 T high-resolution MRI.

## 2. Materials and Methods

### 2.1. Study Population

We enrolled all patients with a diagnosis of DM2 at our diabetes and hypertension outpatient clinic between January 2010 and October 2012. A total of 62 DM2 patients (37 men, 25 women; mean age 56.8 ± 7.5 years) were enrolled in our study. DM2 was defined as repeatedly measured fasting blood glucose ≥ 7.0 mmol/L or nonfasting glucose ≥ 11.1 mmol/L and the test results of glutamic acid decarboxylase antibody (GADA) were negative according to WHO criteria [15]. Diabetes duration was defined as the time from diagnosis of DM2 to MRI scan. All diabetic patients were on treatment with insulin alone or with combined insulin and other antidiabetic drugs. Blood pressure was measured at the time of MRI using a semiautomated sphygmomanometer. Pulse pressure was defined as follows: systolic blood pressure minus diastolic blood pressure. Patient blood was drawn for biochemical tests in the morning after an overnight fast within 2 weeks before MRI. Hypertension (i.e., systolic blood pressure > 140 mmHg or diastolic blood pressure > 90 mmHg or the use of antihypertensive medication [16]), body mass index (i.e., the patient's body weight in kilograms during MR imaging divided by the square root of the height of the patient in centimeters), smoking status (nonsmoker or smoker), and lipid profiles (total cholesterol, high-density lipoprotein, low-density lipoprotein, and triglycerides) were determined. This study was approved by the local medical ethics committee and all subjects gave informed consent to participate in the study.

### 2.2. MRI Protocol

Aortic and brachial artery imaging were performed using a 3.0 T MRI (Signa HDX, GE Medical Systems, Milwaukee, Wis, USA) with ECG-gated technique and an 8-channel phased array body coil. All brain examinations were also performed after arterial imaging using the same MR equipment with an 8-channel phased array head coil. Total imaging time was 45 minutes.

#### 2.2.1. Aortic Arch PWV and Brachial FMD

Aortic arch PWV and brachial FMD were measured using a previously described protocol [12, 13]. Examination parameters are included in Table 1.

**Figure 1:**
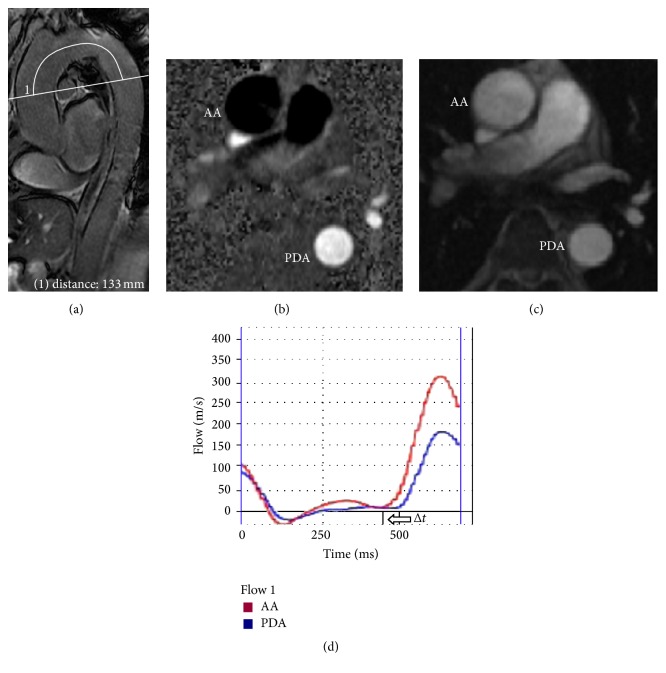
Pulse wave velocity (PWV). (a) Oblique sagittal pilot image of the aorta is used to select the plane at the level of right pulmonary artery and to measure the distance between the ascending aorta (AA) and the proximal descending aorta (PDA) by a series of short straight connected lines along the aortic luminal midline across the aortic arch at the plane. Phase (b) and magnitude (c) images acquired with an electrocardiographically gated gradient echo sequence with velocity encoding at the acquisition sites in the AA and PDA. (d) Sample flow waveforms of the ascending and proximal descending aorta over a cardiac cycle at the plane. Δ*t* is the time between the onsets of the systolic flow waves.

Figure 1 demonstrates assessment of aortic arch PWV between the ascending and the proximal descending aorta. An ECG-gated, spoiled gradient echo sequence with velocity-encoding gradient for phase contrast was applied during breath-hold to the ascending and descending aorta at the level of the right pulmonary artery. Flow waveforms were obtained from the two cross-section planes by using the ReportCARD software on the workstation (GE AW 4.3). The distance (*L*) measurement was done manually by a series of short straight connected lines along the aortic luminal midline across the aortic arch at right pulmonary artery plane and PWV was calculated by dividing the distance around the arch of the aorta by the transit time between the arrivals of the systolic wave front at the two sites.

**Figure 2:**
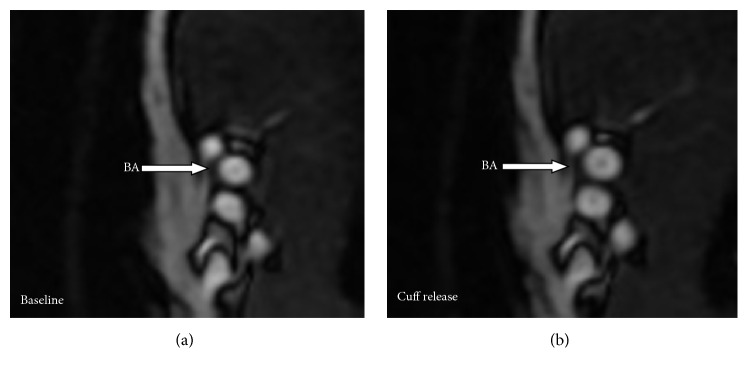
Brachial artery (BA) shown at baseline (a) and after cuff release (b).

Figure 2 demonstrates assessment of FMD. The FIESTA sequence was used. The GE AW 4.3 workstation was used to measure the end diastolic artery cross-section area and to calculate the area change before and after compression. FMD was calculated through the following equation: diastolic function = (area after compression − baseline area before compression)/baseline area before compression × 100%. Maximum percentage change in brachial artery cross-sectional area at end diastole was used to determine the response to the stimulus of cuff compression.

#### 2.2.2. Evaluation of Cerebral Lesions with MRI

For evaluation of cerebral small vessel disease, a spin-echo T2-weighted imaging (T2WI), a fluid-attenuated inversion recovery (FLAIR) imaging, and a T1-weighted gradient echo imaging were performed. Acquisition parameters for T2WI were as follows: TR/TE, 3400/110 ms, flip angle, 90°, field of view: 240 mm, section thickness: 5 mm, gap thickness: 1.5 mm, and 21 sections. Acquisition parameters for the FLAIR were as follows: TR/TE/inversion time (IR): 9000/150/2250, flip angle: 90°, field of view: 240 mm, section thickness: 5 mm, gap thickness: 1.5 mm, and 21 sections. Acquisition parameters for T1WI were as follows: TR/TE, 1750/150, flip angle, 90°, field of view: 240 mm, section thickness: 5 mm, gap thickness: 1.5 mm, and 21 sections.

**Figure 3:**
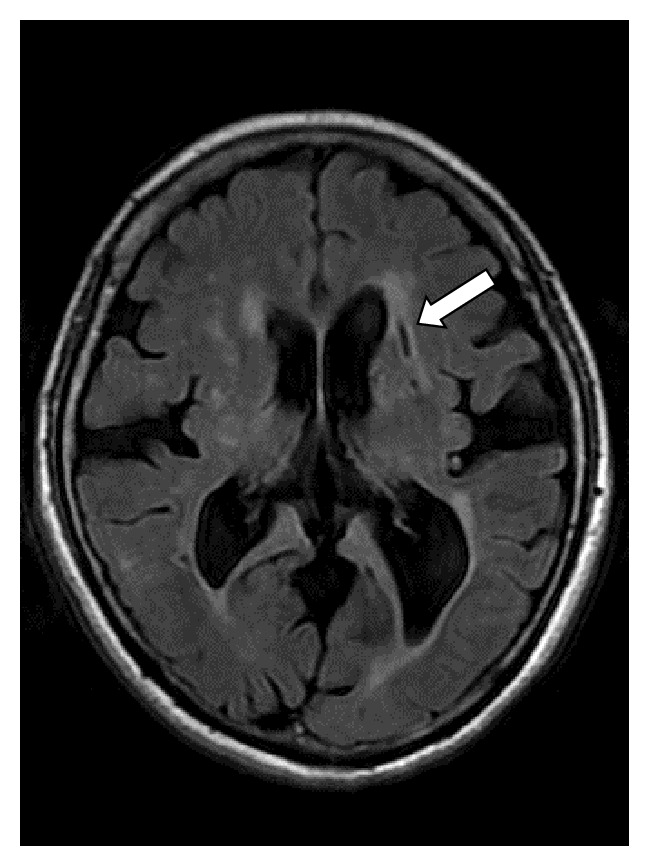
A 60-year-old female patient with type 2 diabetes mellitus for 12 years with lacunar brain infarcts (arrow) on a FLAIR sequence and aortic arch PWV of 10.56 m/s and brachial artery FMD of 13.64%.

**Figure 4:**
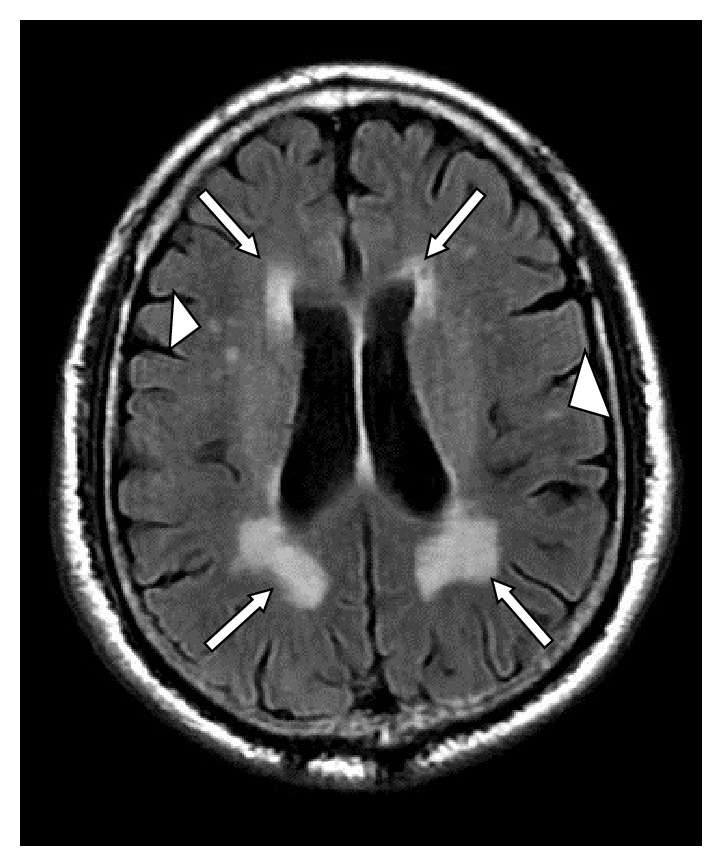
A 68-year-old male patient with type 2 diabetes mellitus for 20 years with abnormal periventricular white matter hyperintensities (WMHs) (arrows) and deep WMHs (arrowheads) on a FLAIR sequence and aortic arch PWV of 7.44 m/s and brachial artery FMD of 5.88%.

For cerebral small vessel disease, two entities including lacunar brain infarcts and white matter hyperintensities (WMHs) were evaluated. Lacunar brain infarcts (Figure 3) were defined as small (but >3 mm in size) cavities within the brain parenchyma, with similar signal intensity to that of cerebrospinal fluid on all pulse sequences, surrounded by an area of high signal intensity on T2 and FLAIR images [17, 18]. Their presence was defined on a binary scale: absent (0) or present (1). WMHs (Figure 4) were defined as areas of brain parenchyma with increased signal on T2-weighted and FLAIR images without mass effect [19]. WMHs were distinguished as either periventricular (pv) WMHs or deep WMHs because of the different pathogenesis involved [18]. WMHs were classified according to Fazekas et al. Fazekas scores of 0 and 1 were considered normal, a score of 2 was considered abnormal below the age of 75 years, and a score of 3 was considered abnormal in any age group [19]. WMHs and lacunar infarcts were visually scored by a consensus reading by two neuroradiologists (10 years of experience in neuroradiology).

### 2.3. Statistical Analyses

All data analysis was performed by an experienced radiologist blinded to the examinees' clinical information. Statistical analyses were carried out using SPSS for windows (version 16.0 SPSS, Chicago, Ill). Data were expressed as mean ± standard deviation unless stated otherwise. Pearson correlation analyses were performed to analyze the association between measured parameters (aortic arch PWV, brachial FMD) and continuous data. Spearman correlation analyses were performed to analyze the association between measured parameters (aortic arch PWV and brachial FMD) and categorical data. Pearson correlation coefficients (*r*), Spearman correlation coefficients (*r*_*s*_), and *P* values were reported. Univariable logistic regression analyses were performed to analyze the association between MRI-measured vascular indices (PWV over aortic arch and FMD) and dichotomous data. Odds ratios (ORs), 95% confidence intervals (CIs), and *P* values were reported. Multiple logistic regression analyses were performed to identify variables that were independently associated with cerebral indices and to adjust for confounders including age, sex, diabetes duration, hypertension, and smoking status.

## 3. Results

### 3.1. Clinical Characteristics of Patients

Table 2 showed the clinical and biochemical characteristics of the study population.

### 3.2. PWV and FMD

Mean PWV over the aortic arch was 6.73 ± 2.00 m/s in DM2 patients. There was a significant correlation between aortic arch PWV and age (*r* = 0.305, *P* < 0.05) and between PWV and hypertension (*r*_*s*_ = 0.498, *P* < 0.001). Sex, smoking status, glycated hemoglobin A1C (HbA1c), heart rate, body mass index, lipid status, and diabetes duration did not correlate with aortic arch PWV.

Mean brachial artery FMD was 16.67 ± 9.11%. FMD was significantly associated with age (*r* = −0.383, *P* < 0.01) and hypertension (*r*_*s*_ = −0.46, *P* < 0.001). FMD was not found associated with sex, smoking status, glycated hemoglobin A1C (HbA1c), heart rate, body mass index, lipid status, and diabetes duration.

### 3.3. Association of Aortic Compliance and Brachial Endothelial Function with Cerebral Lesions

Among 62 DM2 patients, lacunar brain infarcts were diagnosed in 9 patients. Fazekas grade 2 or 3 periventricular WMHs were diagnosed in 18 patients. Fazekas grade 2 or 3 deep WMHs were diagnosed in 28 patients.

Univariable logistic regression analyses showed that aortic arch PWV was associated with lacunar brain infarcts, Fazekas grade 2 or 3 periventricular WMHs, and Fazekas grade 2 or 3 deep WMHs (Table 3). Brachial artery FMD was associated with Fazekas grade 2 or 3 periventricular WMHs and Fazekas grade 2 or 3 deep WMHs (Table 4).

After adjustment for age, sex, smoking status, diabetes duration, and hypertension, aortic arch PWV was significantly associated with lacunar brain infarcts (OR = 2.00; 95% CI: 1.14–3.2; *P* < 0.05), but not with periventricular and deep WMHs. Brachial artery FMD was associated with Fazekas grade 2 or 3 periventricular WMHs (OR = 0.82; 95% CI: 0.71–0.95; *P* < 0.05).

## 4. Discussion

### 4.1. Association between Aortic Compliance and Cerebral Lesions

Applanation tonometry is a well recognized technique for estimating pressure waveforms and arterial stiffness [20]. However, concerning aortic stiffness, this technique can only provide an estimation of PWV along the whole carotid-femoral artery path. Furthermore, tonometry uses body surface measurements to approximate artery length and does not take into account the often torturous route of the vessels. MRI is increasingly used for measuring aortic arch PWV by using accurate aortic length and transit time between flow waves [21, 22]. With the application of three-dimensional (3D) imaging approaches, more parameters including PWV can be measured by MRI and the estimation of arterial stiffness should be accurate and comprehensive [23]. Therefore, PWV measured by MRI was used in our study.

PWV adopted by ESH guidelines is cfPWV (carotid-femoral pulse wave velocity) [24], which is different from that measured by MRI in our study. However, PWV in type 2 diabetes mellitus in our study is higher than that in healthy volunteers measured by the same MR technique [13]. The results are also consistent with the previous report [5]. Therefore, we think that PWV measured by MRI in our study is reliable and can reflect worse diabetic state (HbA1c ~9%) that could suggest target organ damage in the study population. In addition, the techniques used to assess arterial stiffness may not be interchangeable in clinical and research settings and comparisons of findings obtained with different arterial stiffness measures should be conducted with caution [25].

After statistical adjustment for confounding factors of age, sex, smoking status, diabetes duration, and hypertension, our study reveals that aortic arch PWV was significantly associated with lacunar brain infarcts in DM2 patients. Previous studies have shown that DM2 is a powerful risk factor of cardiovascular complications and can make aortic compliance decrease with higher PWV [5, 26]. The association between aortic stiffness and cerebral damage may be indirect with some common vascular risk factors or may be causative in nature [7]. One possible mechanism is that increased aortic stiffness leads to a deficient absorption of the pulse wave and an increase in central pulse pressure, which may augment small vessel disease of the brain through high pulsatile flow. And high pulse pressure blood flow may lead to the brain microvascular damage, including damage to vascular endothelial cells and smooth muscle cells. These situations may cause blood supply to small brain vessels to decrease or interrupt, resulting in cerebral small vessel disease and final strokes [27]. Our results suggest this assumed pathophysiologic mechanism behind the association between aortic stiffness and lacunar brain infarcts in patients with DM2.

Although there are certain common underlying risk factors causing the occurrence of WMHs and cerebral lacunar infarction [28, 29], we found no significant association between aortic arch PWV and WMHs after correction for confounding factors. The possible reason is that the perforating arteries may be affected more than the medullary arteries by reduced aortic compliance which causes the arteriosclerosis in the brain. Damage to the penetrating artery is mostly responsible for lacunar cerebral infarction while medullary artery damage causes WMHs.

### 4.2. Association between Endothelial Function and Cerebral Lesions

Endothelial dysfunction is a systemic disorder, which is characterized by a reduction of the bioavailability of vasodilators, in particular, nitric oxide (NO), and increase of endothelium-derived contracting factors [30]. This imbalance leads to an impairment of the endothelium-dependent vasodilatation, which represents the functional characteristic of endothelial dysfunction. Endothelial dysfunction, which is predisposing to thrombosis, leukocyte adhesion, and smooth muscle cell proliferation, plays a pivotal role in the development, progression, and clinical manifestations of atherosclerosis [31]. Endothelial dysfunction is thought to be one of the important causes of cerebral small vessel disease and an independent predictor of stroke [32, 33]. It has been shown that chronic endothelial dysfunction plays an important role in ischemic leukoaraiosis in cerebral small vessel disease and is related to early deterioration of brain function and poor prognosis of stroke [34]. In addition, vessel injury may cause serious consequences after occurrence of acute ischemic stroke in patients with endothelial dysfunction, due to their decreased vessel protection [8]. WMHs are associated with accelerated stroke and death [35]. Our research showed a significant correlation between FMD and pvWMHs. Therefore, by measuring the brachial artery FMD, we can potentially stratify the risks in DM2 patients. However, no association was found between FMD and deep WMHs and cerebral lacunar infarction. This difference might be attributed to different pathogenic mechanisms behind lacunar infarction, pvWMHs, and deep WMHs.

Our study had some limitations. First, this was a cross-sectional study, and therefore causative mechanisms of cerebral small vessel disease cannot be determined. Furthermore, no age-matched healthy subjects were included to serve as controls. Although renal dysfunction as a risk factor for PWV has been reported in patients with type 1 DM [36], there are no nontraditional risk factors, such as renal function and anemia in our study, which may be due to the small size of sample. However, the primary purpose of this study was to assess the possible relationship between cerebral end-organ damage and aortic compliance and brachial endothelial function in DM2 patients.

In conclusion, aortic arch PWV and brachial FMD measured with high-resolution MRI may be potentially useful to stratify DM2 patients with risk of cerebral small vessel disease.

## Figures and Tables

**Table 1:** MR imaging parameters for assessment of aortic arch PWV and FMD.

Parameter	Aortic arch PWV	FMD
Repetition time (TR)	40.0 ms	3.3~150 ms
Echo time (TE)	5.0 ms	1.5~1.8 ms
Slice thickness	4.0 mm	6.0 mm
Matrix size	256 × 192	224 × 224
Image resolution	1.37 mm × 1.37 mm	1.16 mm × 1.16 mm
Temporal resolution	4.7–7.8 ms	18.75~31.25 ms
Encoding velocity	150 cm/s	/

**Table 2:** Clinical and biochemical characteristics of the study population.

	DM2 patients
	(*n* = 62)
Sex	
Male	37
Female	25
Age (years)	56.84 ± 7.46
Diabetes duration (years)	7.29 ± 5.92
HbA1c (%)	9.65 ± 2.97
Systolic blood pressure (mmHg)	133.06 ± 16.00
Diastolic blood pressure (mmHg)	85.95 ± 10.84
Pulse pressure (mmHg)	47.11 ± 9.73
Heart rate (bpm)	70.48 ± 10.49
Body mass index (kg/m^2^)	24.92 ± 3.95
Smoking	
Yes	46
No	16
Hypertension	
Yes	31
No	31
Cholesterol (mmol/L)	4.87 ± 1.17
HDL (mmol/L)	1.17 ± 0.31
LDL (mmol/L)	2.69 ± 1.01
Triglycerides (mmol/L)	2.36 ± 2.40

Note: values are mean ± SD or data are numbers of patients, DM2: type 2 diabetes mellitus patients, HbA1c: glycated hemoglobin A1C, HDL: high-density lipoprotein, and LDL: low-density lipoprotein. Normal range: systolic/diastolic blood pressure < 140/90 mmHg, heart rate (60–100 bpm), HbA1c (4.0–6.0%), cholesterol (<5.2 mmol/L), triglycerides (0.6–1.7 mmol/L), HDL (>1.04 mmol/L), and LDL (<3.12 mmol/L).

**Table 3:** Association between aortic arch PWV and cerebral lesions.

Parameter	Number of patients	PWV		
		OR	95% CI	*P* value
Lacunar brain infarcts		1.65	1.08–2.53	<0.05
Yes	9			
No	53			
Periventricular WMHs		1.58	1.10–2.27	<0.05
Yes	18			
No	44			
Deep WMHs		1.53	1.07–2.17	<0.05
Yes	28			
No	34			

**Table 4:** Association between brachial artery FMD and cerebral lesions.

Parameter	Number of patients	FMD		
		OR	95% CI	*P* value
Lacunar brain infarcts				0.22
Yes	9			
No	53			
Periventricular WMHs		0.88	0.80–0.97	<0.01
Yes	18			
No	44			
Deep WMHs		0.91	0.85–0.98	<0.05
Yes	28			
No	34			
